# Identification of copy number variations and common deletion polymorphisms in cattle

**DOI:** 10.1186/1471-2164-11-232

**Published:** 2010-04-09

**Authors:** Joon Seol Bae, Hyun Sub Cheong, Lyoung Hyo Kim, Suk NamGung, Tae Joon Park, Ji-Yong Chun, Jason Yongha Kim, Charisse Flerida A Pasaje, Jin Sol Lee, Hyoung Doo Shin

**Affiliations:** 1 Laboratory of Genomic Diversity, Department of Life Science, Sogang University, Shinsu-dong, Mapo-gu, Seoul 121-742, Republic of Korea; 2 Department of Genetic Epidemiology, SNP Genetics, Inc., Room 1407, Complex B, WooLim Lion's Valley, 371-28, Gasan-Dong, Geumcheon-Gu, Seoul 153-801, Republic of Korea

## Abstract

**Background:**

Recently, the discovery of copy number variation (CNV) led researchers to think that there are more variations of genomic DNA than initially believed. Moreover, a certain CNV region has been found to be associated with the onset of diseases. Therefore, CNV is now known as an important genomic variation in biological mechanisms. However, most CNV studies have only involved the human genome. The study of CNV involving other animals, including cattle, is severely lacking.

**Results:**

In our study of cattle, we used Illumina BovineSNP50 BeadChip (54,001 markers) to obtain each marker's signal intensity (Log R ratio) and allelic intensity (B allele frequency), which led to our discovery of 855 bovine CNVs from 265 cows. For these animals, the average number of CNVs was 3.2, average size was 149.8 kb, and median size was 171.5 kb. Taking into consideration some overlapping regions among the identified bovine CNVs, 368 unique CNV regions were detected. Among them, there were 76 common CNVRs with > 1% CNV frequency. Together, these CNVRs contained 538 genes. Heritability errors of 156 bovine pedigrees and comparative pairwise analyses were analyzed to detect 448 common deletion polymorphisms. Identified variations in this study were successfully validated using visual examination of the genoplot image, Mendelian inconsistency, another CNV identification program, and quantitative PCR.

**Conclusions:**

In this study, we describe a map of bovine CNVs and provide important resources for future bovine genome research. This result will contribute to animal breeding and protection from diseases with the aid of genomic information.

## Background

Cattle have been important to human culture for over 8,000 years as an agricultural means, for transportation, and as a supply of meat and milk [1]. In recent years, studies have been conducted that attempt to increase the productivity of meat and marbling grades by utilizing genetic factors [2-5], and the results of these studies have been deemed economically significant. The bovine genome is made up of 29 autosomes and sex chromosomes with a genome size estimated to be around 2.87 Gbp. Because of the economic importance of cows, the Bovine Genome Sequencing and Analysis Consortium has decoded bovine whole-genomic information (Bovine Genome Project) and has reported that a minimum of 22,000 genes are included in the cattle genome [6]. These findings show that bovine genome analysis is becoming increasingly popular.

Copy number variation (CNV) is an event in which a large DNA fragment (> 1 kb) is duplicated or deleted. According to recent studies, structural variations that include CNV affect gene expression and are related to the onset of many diseases [7-10]. However, these studies usually focused only on the human genome, while studies of other animals such as cows have been minimal. Although a recent study found 25 CNVs in three Holsteins by array comparative genomic hybridization (aCGH) [11], an analysis that uses many bovine samples to find a way to utilize the cow's genomic character economically is yet to be conducted. Moreover, CNVs in genomes exist in low frequency; therefore, it is advisable to analyze many samples in order to find common CNV regions for analysis with various phenotypes. In the case of animal genomes, Skinner and colleagues have reported a detailed molecular cytogenetic map as a result of a comparative genomics study in chicken and Pekin duck using a CGH microarray [12], and Griffin and colleagues also reported 16 interspecific CNVs between chicken and turkey [13]. As in the case of cattle genome, a recent paper reported 25 CNVs discovered using array comparative genomic hybridization (aCGH) with 3 Holsteins as samples [11]. However, in order to investigate the association between various economically beneficial phenotypes and CNVs, more bovine CNVs would need to be discovered.

Two platforms for identifying individual CNVs, aCGH and a single nucleotide polymorphism (SNP) genotyping array, have been widely used. In the case of the former, signal intensity was varied when comparing the reference and target with the dye-swap method [8]. Regarding the latter, clustered pool references, signal intensity, and allelic intensity (B allele frequency) were used to identify CNV [14-16]. The SNP genotyping array has the advantage of performing both whole-genome SNP association analysis and CNV analysis [14]. This platform also provides various information including Mendelian inconsistency (heritability error), deviation from Hardy-Weinberg equilibrium (HWE), and genotype missing rate. Recent studies have used this advantage to identify common deletion polymorphisms efficiently [17-19]. In addition, accurate and efficient algorithms have also been developed recently that discover CNV not only by means of signal intensity, but also through B allele frequency and family information, and these methods are widely used [15,20-24]. In order to detect reliable CNVs, we studied multiple factors including signal intensity, B allele frequency, marker distance, and population frequency of the B allele (PFB) using PennCNV [9,24,25].

In this study, we examined 256 bovine samples using a SNP genotyping array to discover genomic variations that include individual CNVs and common deletion polymorphisms from the whole cattle genome. Our goal is to provide genomic variation information that could be used to find economical genetic traits in cattle.

## Results

**Figure 1:**
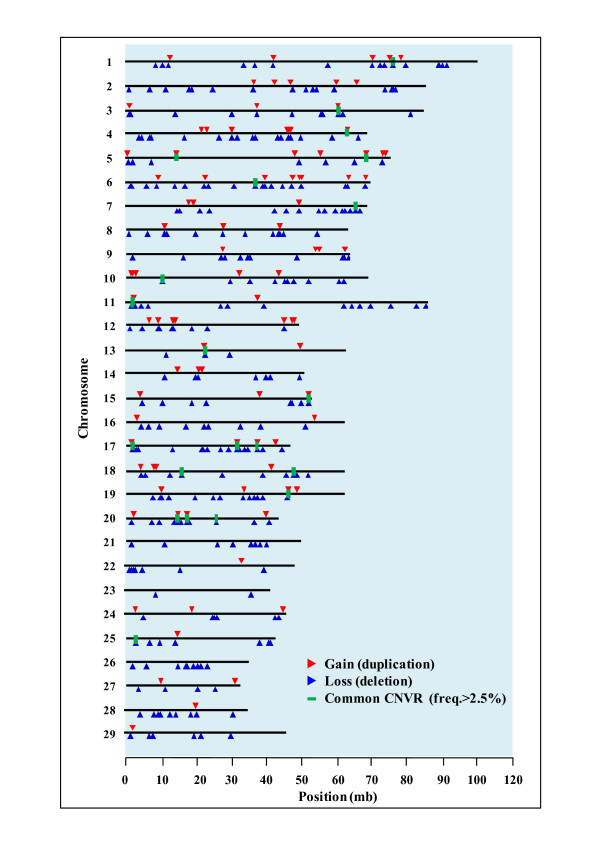
**Map of identified copy number variations in *Bos taurus coreanae***. The locations of all copy number variation regions (CNVRs) are depicted by triangles (red color: gain; blue color: loss). The thick line (color: green) indicates common CNVRs (freq. > 2.5%).

In this study, we used Illumina BovineSNP50 BeadChip and PennCNV to identify CNVs in cattle (Additional file 1; table s1). One sample contained an average of 3.2 CNVs with an average length and a median size of 149.8 kb and 118.7 kb (Table 1), respectively. After all CNVs were aggregated for the CNV region (CNVR), 368 CNVRs were identified (Additional file 2; table s2). The average number of CNVs per sample was 1.4, with an average length and median size of 171.5 kb and 128.3 kb, respectively. Furthermore, 76 CNVRs with > 1% frequency, 22 CNVRs with > 2.5% frequency, and 6 CNVRs with > 5% frequency were also inferred from this study. A total of 538 genes were included in the identified CNVR (Table 1). Common CNVRs with CNV frequency higher than 2.5% are listed in Table 2, with chr15:1836732-2039483 CNVR having the highest frequency (13.2%) and chr17:75520590-76487768 CNVR having the highest number of genes. Figure 1 shows the map of CNVRs discovered from this study. We were also able to detect 368 CNVRs that were distributed evenly across the chromosomes. Among them, we found 99 CNVRs with only gain (duplication), 310 with only loss (deletion), and 22 CNVRs (freq. > 2.5%) that share common values.

**Table 1:** Summary of identified copy number variations in *Bos taurus coreanae *(*n *= 265)

	Total number	Average no. of CNVs per sample	Average size of CNVs (kb)	Median size of CNVs (kb)	No. of Gain	No. of Loss	Ratio (Loss/Gain)	No. of common CNVs (freq. >1%)	No. of common CNVs (freq. >2.5%)	No. of common CNVs (freq. >5%)	Genes
Individual CNV	855	3.2	149.8	118.7	221	634	2.9	-			-

CNV region	368	1.4	171.5	128.3	-	-	-	76	22	6	538

**Table 2:** Summary of common copy number variation regions in *Bos taurus coreanae *(freq. >2.5%)

CNV region	Length (bp)	No. of CNVs	Frequency	No. of genes	Genes
chr15:1836732-2039483	202,751	35	0.132	0	
chr5:11483310-11889745	406,435	28	0.106	0	
chr17:75520590-76487768	967,178	24	0.091	21	*ARVCF, C17H22orf25, CDC45L, CLDN5, COMT, DGCR14, DGCR2, DGCR8, FAM128B, LOC515651, MED15, PI4KA, RANBP1, SEPT5, SERPIND1, SLC25A1, SNAP29, THAP7, TUBA3E, TXNRD2, UFD1L*
chr17:15002419-15372017	369,598	22	0.083	1	*SMARCA5*
chr13:54700988-55222116	521,128	16	0.060	12	*ARFGAP1, ARFRP1, C13H20ORF11, C13H20orf149, C13H20orf195, DIDO1, EEF1A2, RTEL, SLC17A9, STMN3, TNFRSF6B, ZGPAT*
chr20:31559229-31832019	272,790	15	0.057	0	
chr3:36163190-36338393	175,203	13	0.049	2	*CSF1,GSTM3*
chr4:10009287-10665698	656,411	13	0.049	3	*GATAD1, LOC524650, MGC148329*
chr5:102164053-102261488	97,435	10	0.038	2	*GUCY2C, PLBD1*
chr19:12442436-12611334	168,898	10	0.038	0	
chr11:109101259-109497448	396,189	9	0.034	10	*C11H9ORF142, C8G, CLIC3, EDF1, KIAA1984, MAMDC4, PARF, PHPT1, PTGDS, TMEM141*
chr18:10398490-10604602	206,112	9	0.034	1	*MGC140224*
chr18:48593919-48725107	131,188	9	0.034	6	*EID2, MED29, RPS16, SUPT5H, TIMM50, ZFP36*
chr20:46603190-46767627	164,437	9	0.034	0	
chr25:42346692-42719563	372,871	9	0.034	3	*CARD11, CHST12, LFNG*
chr1:40050487-40150878	100,391	8	0.030	0	
chr5:123127110-123347200	220,090	8	0.030	1	*PPARA*
chr7:4650135-5033417	383,282	8	0.030	11	*CIST1, IFI30, ISYNA1, JUND, LRRC25, LSM4, MPV17L2, PGPEP1, PIK3R2, RAB3A, SSBP4*
chr17:24499559-24727631	228,072	8	0.030	0	
chr20:51402609-51459233	56,624	8	0.030	0	
chr6:56495043-56634157	139,114	7	0.026	0	
chr10:90756777-90887327	130,550	7	0.026	0	

**Figure 2:**
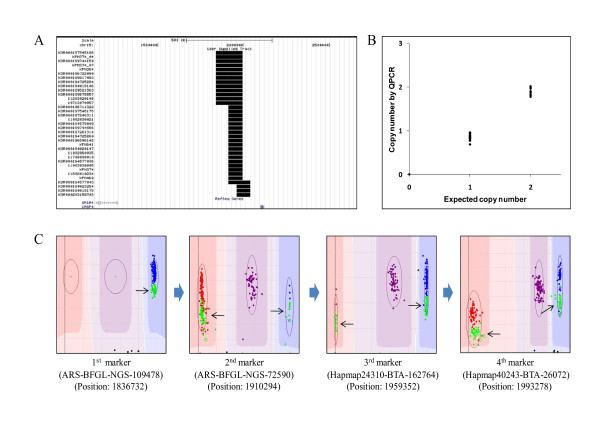
**Visualization and validation of copy number variation region (chr15:1836732-2039483) by visual examination and quantitative PCR**. (A) Visualization of identified individual copy number variations in UCSC Genome Browser. The black bars indicate copy number variation of each sample. (B) Determination of copy number by quantitative PCR around third marker (Hapmap24310-BTA-162764). (C) Visual examination by consecutive genoplot images of markers. The first marker shows a monomorphic pattern having 2× (color: blue), 1× (color: cyan), and 0× (color: black). The samples having a deletion (copy number: 1×; color:cyan) were consecutively displayed in deletion position to the fourth marker.

**Figure 3:**
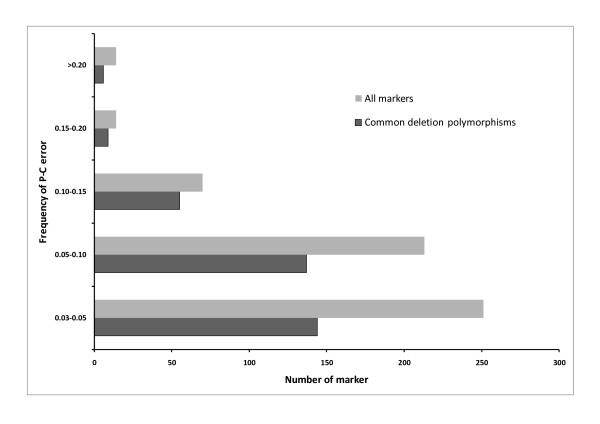
**Distribution of identified common deletion polymorphisms according to the frequency of parent (sire)--child (steer) heritability (P-C) error**.

**Figure 4:**
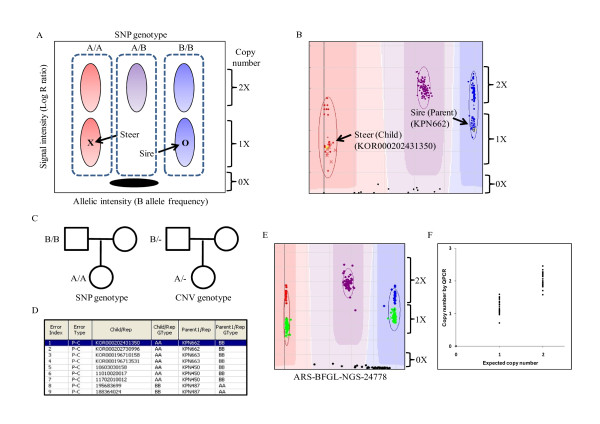
**Scheme of identification of common deletion polymorphisms by parent (sire)--child (steer) heritability (P-C) error and validation by quantitative PCR**. (A) Scheme of genoplot image in Illumina BeadStudio 3.2 software where SNP marker was located within copy number variation region. Steer and sire were marked as "X" and "O", respectively. The three dotted lines represent three SNP genotypes (A/A, A/B, and B/B). (B) Genoplot image showing P-C heritability error. The steer (child) is shown on the left side (copy number: 1×; CNV genotype: A/-; marked at "X") and its sire (parent) on the right (copy number: 1×; CNV genotype: B/-; marked at "O"). (C) Difference of SNP and CNV genotype in one pedigree. (D) Heritability error table. Nine P-C errors in one marker are displayed. The table shows the sample ID of steer (child) and sire (parent) and their SNP genotypes having heritability error. (E) Genoplot image of identified common deletion polymorphisms (marker name: ARS-BFGL-NGS-24778). Three types of copy number (2×, 1×, and 0×) are depicted. Individuals having hemizygous deletions (copy number: 1×) clustered into two distinct groups (color: cyan). Samples having null copy number are displayed with a black dot at the bottom. (F) Validation by qPCR around the ARS-BFGL-NGS-24778 marker. The individuals having homozygous (null) and hemizygous deletions in the genoplot image were spotted approximately in the same copy number position by qPCR.

The sizes of the identified CNVs ranged from 50~200 kb, with a few outliers having a size of 250 kb, and most chromosomes had more loss than gain (Additional file 3; figure s1, s2). Figure 2 shows the result of visual examination by genoplot image within chr15:1836732-2039483 and validation by qPCR. Samples representing hemizygous deletion (color: cyan; copy number: 1×) of the first marker had the same intensity position up to the fourth marker, consecutively (Figure 2c). The real copy numbers of samples by qPCR around the third marker (marker name: Hapmap24310-BTA-162764; position: 1959352) were matched with expected copy numbers on the genoplot image (Figure 2b). In addition, most identified CNVs using CNVPartition overlapped with the CNVs detected using PennCNV (94%) (Additional file 4; table s3). To identify common deletion polymorphisms, we used two methods: a heritability error analysis called genotype transmission error, and pairwise analysis. In order to analyze heritability error, 156 sire and steer family sets were used. These sets had parent-child heritability frequency that was equal to or greater than 99.6% and a confirmed parent-child relationship. To calculate for heritability error, we used Illumina BeadStudio 3.2 software and detected a total of 990 errors. From that, we selected 320 parent-child heritability errors whose frequency was equal to or greater than 3% for the purpose of identifying common deletion polymorphisms. Figure 3 displays a number of identified common deletion polymorphisms for each frequency of father (sire) and child (steer) heritability error. When the number of heritability errors increased, the number of distributed markers decreased. However, there were more common deletion polymorphisms (71.4%) observed for markers with higher heritability frequency (> 10%) than for those (60.6%) with low heritability frequency (<10%). Following this method, we found a total of 351 common deletion polymorphisms. Moreover, we were also able to detect 192 common deletion polymorphisms by pairwise comparison, analyzing between reference and target samples. Merging the identified common deletion polymorphisms from the two methods, we were able to identify a total of 448 common deletion polymorphisms, with 95 of them common to both methods (Additional file 5; table s4). Common deletion polymorphisms found in this study were distributed from chromosomes 1 to 29 quite evenly, with chromosome 2 having the most and chromosome 25 having the least (Additional file 3; figure s3). In order to quantitatively measure for common deletion polymorphisms, we performed qPCR around ARS-BFGL-NGS-24778 in chromosome 2 (Position: 61648422) (Figure 4e). As a result, we were able to confirm that the expected copy number changes in each sample, based on visual examination of the genoplot image, existed at those sites (Figure 4f).

## Discussion

Cattle are important resources for humans as providers of meat and milk and as labor power for agriculture. Lately, interest and research concerning bovine genetic resources are increasing, as evident in the completion of the Bovine Genome Project. However, current studies on bovine CNV, which is an important area of genetic variation along with SNPs, are very minimal. In the past few years, CNVs have been studied extensively in the human genome, and many human CNVs have been discovered and reported to the DGV (Database of Genomic Variants; http://projects.tcag.ca/variation). Recently, identifying how CNVs in the human genome affect various given phenotypes, including disease susceptibility, has become a major focus for researchers [26]. In the animal genome, certain economically useful phenotypes undoubtedly exist. Based on the findings from this study, future research might be able to examine the genetic effects of CNV on various economic traits, including beef quality. In order to facilitate such studies, CNVs discovered from each animal should be entered in a database similar to that of the human genome. Until now, most CNV researchers have run their association analysis using CNV genotyping according to differences in signal intensity alone. However, CNV is usually linked to nearby SNPs. Just lately, CNV and SNP combined analysis has been used. The main advantage of this method is that it can analyze signal intensity and allelic differences simultaneously. In other words, it is possible to do multi-allelic CNV/SNP genotyping on CNVs containing multiple SNP markers. Our previous study reported that after discovering and genotyping multi-allelic CNV markers in the deletion region of the human genome, CNV/SNP combined analysis provided more reliable association results than using SNP or CNV genotyping alone [27].

In this study, we identified 855 bovine CNVs and 368 CNVRs. To apply the findings of this study, the common CNVRs we were able to identify will be useful in analyzing certain relationships among phenotypes. For example, a common CNVR provides important genome information for discovering genes related to beef grade and meat productivity. CNV identification was performed using Illumina BeadChip and algorithm made from Btau_4.0. If UMD3 bovine genome assembly was used, more accurate CNV identification result would have been expected. Identified CNVRs in this study were validated using visual examination, in which comparison with the results of CNVPartition and qPCR was performed. In Figure s4 (additional file 6), we demonstrate how this value changes in a region where copy number change appears in a schematic way. For a normal copy number (2×), two homozygous genotypes (A/A and B/B) and one heterozygous genotype (A/B) appear on one genoplot image. On the other hand, a heterozygous genotype (A/B), which only emerges in a normal copy number (2×), disappeared in a region where deletion (1×) occurred. This explains why only two lines (A/A and B/B) were evident. If gain (copy number: 3×) appears in the genoplot image, there would have been four lines (A/A/A, A/A/B, A/B/B, and B/B/B). If B allelic frequency and signal intensity were simultaneously used to discover CNV in the case of deletion and duplication, the identification would be more accurate. To date, the study by Liu et al. is the only one regarding bovine CNV identification [11], as far as we know, and it reported 25 CNVs from three Holstein using array CGH (Btau 3.0). To compare with previous CNV data on cattle, we converted the coordinates of the 25 bovine CNVs with Btau4.0 using the LiftOver tool in the UCSC database. However, only one CNV overlapped with our results. Although it is not easy to decipher this discrepancy, different breeds of cattle and/or a smaller number of animals was used in the previous study.

CNV is defined as a DNA fragment higher than 1 kb, and copy number variation smaller than 1 kb is called an indel. Recently, it has been reported that the latest discoveries of CNV sizes were much smaller than the previous results due to the advances made in the chip platform [17,28]. Zhang and colleagues have mentioned that the cutoff value of 1 kb is completely arbitrary, and they suggest choosing an average exon size (~100 bp) in defining CNV [26]. Also, Venter and Watson demonstrated that CNV size distributions show a marked enrichment ranging from 300 to 350 bp using whole-genome shotgun sequencing and massively parallel DNA sequencing methods [29,30]. Although we used a 50K chip for this study, high-resolution methods used for human genome study such as high-density chip or next generation sequencing should be applied to animal genomes, including cattle. For future studies determining the exact CNV boundary, this current study would be valuable in that it could serve as a preliminary report providing whole-genome CNV distribution resources regarding the cattle genome.

Indels could affect phenotype and gene expression dosage such as CNV, and may need to be studied further. We developed a method to efficiently discover common deletion polymorphisms among indels, and subsequently identified 448 common deletion polymorphisms in this study. Figure 4 schematically shows the cause of sire and steer heritability error when an SNP marker exists within the CNVR. If a deletion occurs on an allele, subsequently it will lead to heritability errors since if a sample exists on the 1× position, the SNP genotype becomes A/A (steer) and B/B (sire), as exhibited in Figure 4c. Using this heritability error, investigation of a region with frequent P-C errors can increase the accuracy and efficiency of identifying the variants. This method is much more effective than the one previously used [27].

Gene ontology (GO) analysis can provide insight into the functional enrichment of CNVs. For this reason, we ran GO analysis using DAVID http://david.abcc.ncifcrf.gov provided by the National Institute of Allergy and Infectious Diseases (NIAID) and NIH [31]. As a result, we found that genes significantly enriched in the identified bovine CNVs include the cytoplasm, intracellular part, cytoplasmic part, and intracellular organelle. Since CNV can influence regions within 500 kb, we performed additional enrichment analysis. The gene functions enriched in nearby genes include the multicellular organismal process, regulation of biological quality, and cell morphogenesis (Table 3). This analysis provides estimated results of expected functions, so additional study of function consequences between actual phenotypes should be carried out.

**Table 3:** Gene ontology (GO) categories significantly overrepresented in bovine copy number variations

Group	GO Term	Count	*P*-value
Gene	cytoplasm	73	7.62E-10
	intracellular part	95	7.74E-09
	cytoplasmic part	52	9.79E-08
	intracellular organelle	81	3.74E-07
	organelle	81	3.74E-07
	intracellular	100	3.76E-07
	developmental process	31	2.14E-06
	intracellular membrane-bound organelle	70	3.87E-06
	membrane-bound organelle	70	3.93E-06
	binding	130	5.64E-06
	cell differentiation	22	1.70E-05
	cellular developmental process	22	1.70E-05
	negative regulation of cellular process	16	2.27E-05
	multicellular organismal development	22	4.24E-05
	regulation of apoptosis	12	4.28E-05
	regulation of programmed cell death	12	4.87E-05
	negative regulation of biological process	16	5.02E-05
	intracellular organelle part	40	5.89E-05
	cell development	17	6.14E-05
	organelle part	40	6.36E-05
	protein binding	61	8.41E-05
	apoptosis	14	1.06E-04
	programmed cell death	14	1.17E-04
	biological regulation	47	1.72E-04
	organelle membrane	22	1.90E-04
	regulation of cellular process	41	2.02E-04
	death	14	2.37E-04
	cell death	14	2.37E-04
	regulation of biological process	43	2.62E-04
	DNA replication	8	2.67E-04
	multicellular organismal process	25	6.73E-04
	calmodulin binding	6	7.07E-04
	anatomical structure development	17	8.08E-04
	cell cycle	12	9.23E-04

Nearby gene	multicellular organismal process	19	6.90E-07
	regulation of biological quality	11	2.98E-05
	cell morphogenesis	6	5.63E-04
	cellular structure morphogenesis	6	5.63E-04
	cellular morphogenesis during differentiation	4	7.67E-04

This study aims to provide genomic resources required for analyzing what economic impact phenotypes and bovine CNVs can bring to the table. In the case of the human genome, the map of identified CNVs presented by Redon and colleagues [8] is now used as important information in association studies on various diseases including autism, inflammatory autoimmune disorders, idiopathic learning disability, lung cancer, systemic lupus erythematosus, and schizophrenia. In addition, follow-up studies for high-resolution CNV mapping have been actively occurring. However, CNVs related to economically useful phenotypes are yet to be thoroughly researched, so we expect the results of this study to provide meaningful genomic variation information for related research.

Future studies should include additional analysis for accurate size estimation of bovine CNVs and common deletion polymorphisms found in this study, followed by an association analysis of bovine phenotypes.

## Conclusions

In summary, we have identified 855 new CNVs and 448 common deletion polymorphisms in *Bos taurus coreanae*. These variations were successfully validated using visual examination, Mendelian inconsistency, CNVPartition, and qPCR. Here, we report the map of bovine CNVs. We expect this result will provide important resources for future bovine genome research.

## Methods

### Subjects and Illumina Infinium II assay

The cattle (*Bos taurus coreanae*) genomic DNA samples were obtained from 248 steers produced from 17 sires (*n *= 265). All blood samples were collected from the Seosan Livestock Institute (NLRI). We used the Illumina BovineSNP50 BeadChip containing 54,001 markers that uniformly span the entire bovine genome (Illumina, Inc., USA). Those markers were obtained by Illumina's Genome Analyzer, a next-generation sequencing system, and publicly available sources including a bovine reference genome (Btau 4.0) and Bovine HapMap Consortium data set. The mean and median of spacing in this BeadChip was 51.5 kb and 37.3 kb, respectively. Using Illumina's Infinium II assay, the genotyped data for a total of 54,001 markers were collected for identification of bovine CNVs. The assay procedure used has been described in our previous study [27]. We incorporated single-base extension (SBE) which uses a single probe sequence that is 50 bp long and is designed to hybridize immediately adjacent to the SNP query site. Briefly, each sample was whole-genome amplified, fragmented, precipitated, and re-suspended in an appropriate hybridization buffer. Denatured samples were hybridized on the BovineSNP50 BeadChip for a minimum of 16 h at 48 degree. After completion of the assay, the BeadChips were scanned with a two-color, confocal BeadArray reader. Scanned image intensities were loaded directly into Illumina's BeadStudio 3.2 software. When normalization was completed, the clustering process was performed to assess cluster position for each marker and to determine individual genotypes. The overall SNP genotyping call rate was 99.57%, which indicated that high-quality data was extracted for this study.

### Identification of bovine copy number variations

All signal intensity (log R ratio: LRR) and allelic intensity (B allele frequency: BAF) ratios of samples were reported using Illumina BeadStudio 3.2 software. We used high quality samples with a standard deviation (SD) of LRR < 0.30 to assess the noise of the intensity signal. To identify individual CNVs, we used the PennCNV, which incorporates multiple factors including LRR, BAF, marker distance, and population frequency of the B allele [15,32]. Because bovine has 29 autosomal chromosomes, we used an alternative program argument; the "-lastchr 29" in the "-detect" argument to be specific CNV regions (CNVRs) were aggregated from identified CNVs by considering each other's overlapping regions. For verification purposes of the identified CNVs, CNVpartition program with default criteria (Illumina Inc., USA) was initially used to identify CNVs after which, results were compared with those that were obtained using PennCNV.

### Identification of common deletion polymorphisms

If a deletion is positioned on the SNP marker, deviation from Hardy-Weinberg equilibrium, Mendelian inconsistency (heritability error) in a family (Figure 4), and a high missing genotype rate particularly appear. To identify common deletion polymorphisms in this study, we used heritability error of the genotype for both sire (parent) and steer (child). In our previous study, we found that common deletion polymorphisms have a unique pattern in the BeadStudio genoplot [27]. We designated these common deletion polymorphisms to multi-allelic CNV markers because they had six distinct genotypes (A/A, A/B, B/B, A/-, B/-, and -/-) according to the differences in the copy numbers and allelic intensities [33]. Recently, other research has also described the two-dimensional feature of each marker in an SNP genotyping array [9,16,34]. To identify common deletion polymorphisms, we selected candidate markers with a frequency of sire and steer heritability error (P-C error) > 3%. After we checked the heritability of 156 sire and steer sets, heritability error frequency was calculated using BeadStudio 3.2. Among the candidate markers, we found common deletion polymorphisms representing six distinct cluster images by visual inspection. In addition, we used the pairwise method for identifying hidden common deletion polymorphisms. After selecting a high-quality reference sample, we constructed paired sets representing intensity differences between target and reference samples using the paired sample editor in BeadStudio 3.2. The range of inspection of the marker was both log_2_(R_sub_/R_ref_) ≤ -1.5 and log_2_(R_sub_/R_ref_) ≥ 1.5 (Additional file 3; figure s5).

### Validation by quantitative PCR

To validate the existence of both the CNV region and common deletion polymorphisms, we performed TaqMan real-time PCR on an ABI Prism 7900 sequence detection system (Applied Biosystems, Foster City, CA). Specific probes were generated by Primer Express 2.0 (Additional file 6; table s5). The basic transcription factor 3 (*BTF3*) gene, which served as an internal standard, was co-amplified with the marker. Amplification reactions (10 ul) were carried out in duplicate with 10 ng of template DNA, 1× TaqMan Universal Master Mix buffer (Applied Biosystems), 900 nM of each primer, and 250 nM of each fluorogenic probe. Thermal cycling was initiated with a 2-min incubation at 50 degrees followed by a denaturation step from 10 min at 95 degrees, to 40 cycles of 15 sec at 95 degrees, and lastly to 1 min at 60 degrees. Three replicate reactions were performed for primer pairs, and a comparative C_T _method was used to calculate the copy number [35] (Applied Biosystems user bulletin #2 [P/N4303859]). ΔC_T _was calculated by subtracting the *BTF3 *C_T _value from the sample C_T _value for each replicate. The average C_T _value for the three replicates was then calculated. In order to determine the ΔΔC_T_, ΔC_T_'s from all other samples were normalized. Finally, the copy number was given using the formula 2 × 2^-ΔΔCT^.

### Validation by visual examination of genoplot image

Illumina BeadStudio 3.2 software provides visual genoplot images representing signal intensity (Y-axis) and allelic intensity (X-axis) simultaneously per marker. To validate the existence of both identified CNVRs and common deletion polymorphisms in this study, we visually inspected the consecutive changes in signal intensity and allelic intensity for each sample at each genoplot image using the above software.

## Authors' contributions

J.S.B. performed microarray data analysis, produced the results and drafted the manuscript. H.S.C., L.H. K. and S. N. contributed to the preparation of samples and Infinium II assay. T.J.P., J.-Y.C. and J.S.L. provided technical assistance. J.Y.K. and C.F.A.P. have been involved in critically revising the manuscript for important intellectual content. H.D.S. contributed to the conception of the study and participated in the interpretation of the results and revision of the manuscript. All authors read and approved the final manuscript.

## Supplementary Material

Additional file 1 Supplementary Table S1. Click here for file

Additional file 2 Supplementary Table S2. Click here for file

Additional file 3 Supplementary Figures. Click here for file

Additional file 4 Supplementary Table S3. Click here for file

Additional file 5 Supplementary Table S4. Click here for file

Additional file 6 Supplementary Table S5. Click here for file
