# Supplementary material for: Identification of copy number variations and common deletion polymorphisms in cattle
Source: BMC Genomics. 2010 Apr 9;11:232. doi: 10.1186/1471-2164-11-232 (PMC2859865; doi:10.1186/1471-2164-11-232)
Supplement: Additional file 3 — Supplementary Figures. [file 1471-2164-11-232-S3.DOC]

**Supplementary Figure legends:**

**Supplementary Figure 1. Distribution of identified copy number variations in chromosomes**

**Supplementary Figure 2. Size distribution of identified copy number variations and aggregated copy number variation regions in *Bos taurus coreanae***

**Supplementary Figure 3. Distribution of identified common deletion polymorphisms in chromosomes**

**Supplementary Figure 4. Two-dimensional positioning of SNP marker within CNV region**

A. Two-dimensional genoplot image representing multi-allelic CNV genotypes in all samples. Nine distinct CNV genotypes are displayed according to their signal intensity (Y-axis) and allelic intensity (X-axis). B. B-allelic frequency plot of one sample. The B-allelic signal values of normal copy number (2X), hemizygous deletion (1X), and duplication (3X) are clearly separated into three kinds of pattern. The application of B-allelic signals for identifying individual CNVs provides more accuracy than only using signal intensity.

**Supplementary Figure 5. Identification of common deletion using pair-wise method**

Supplementary Figure 1


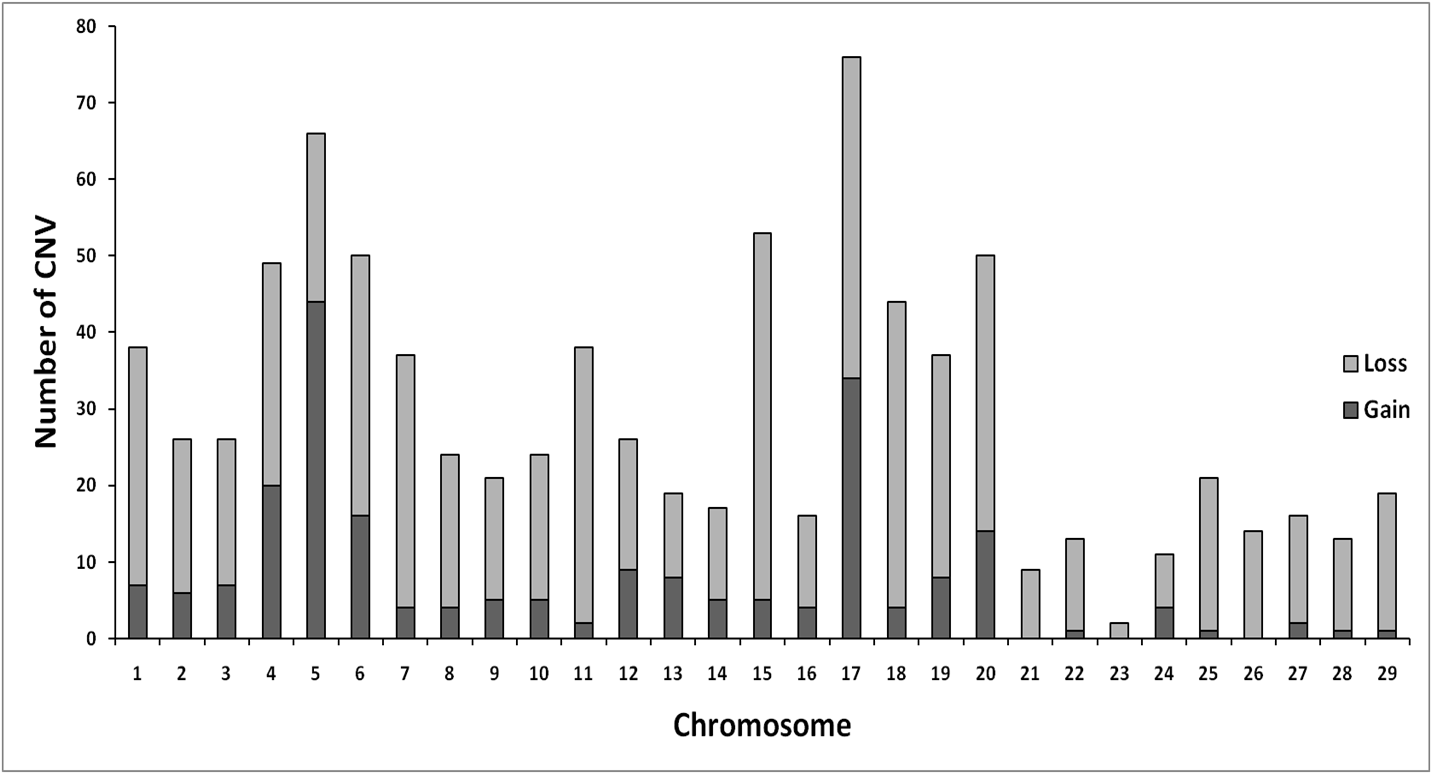


Supplementary Figure 2


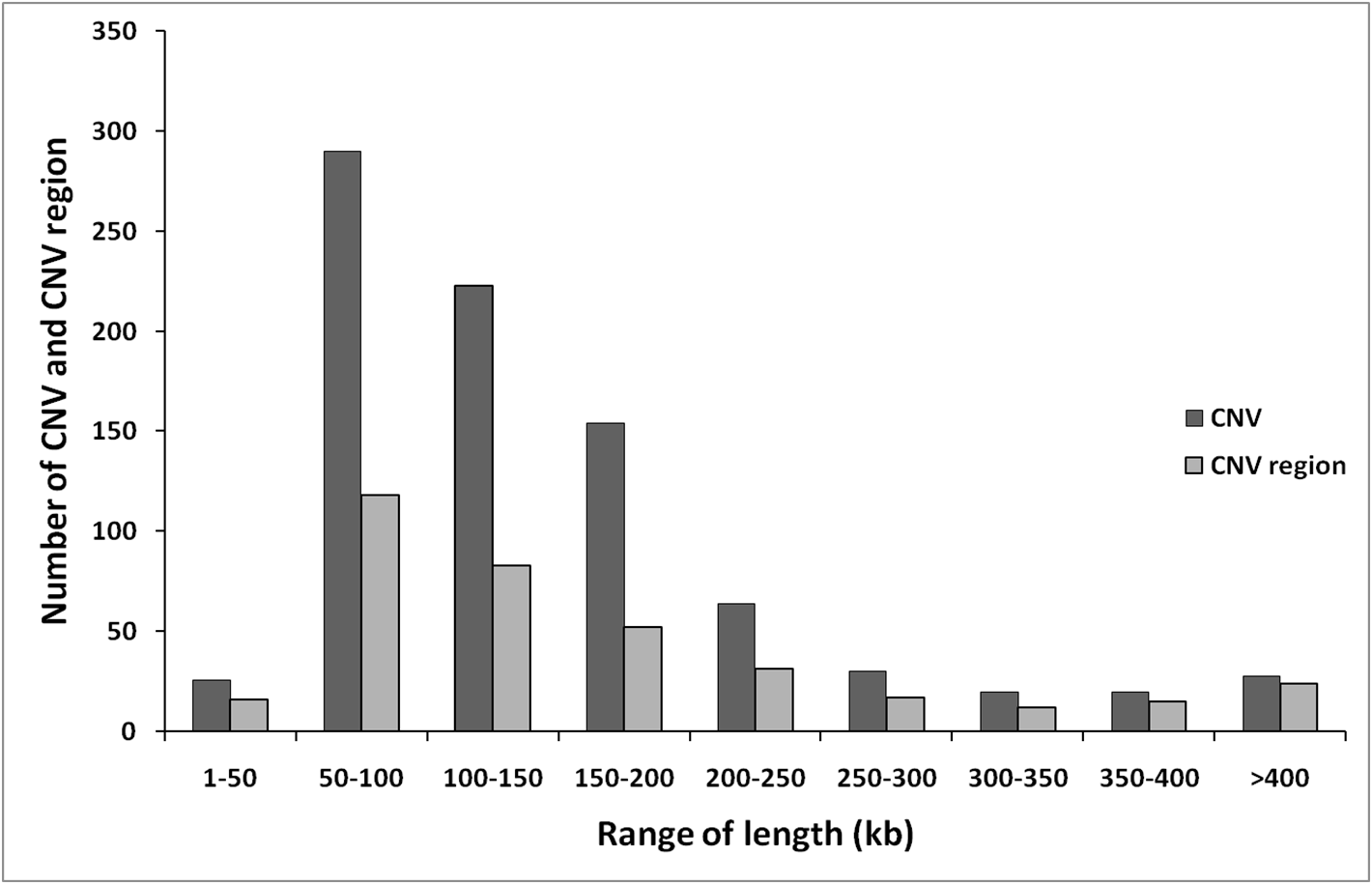


Supplementary Figure 3


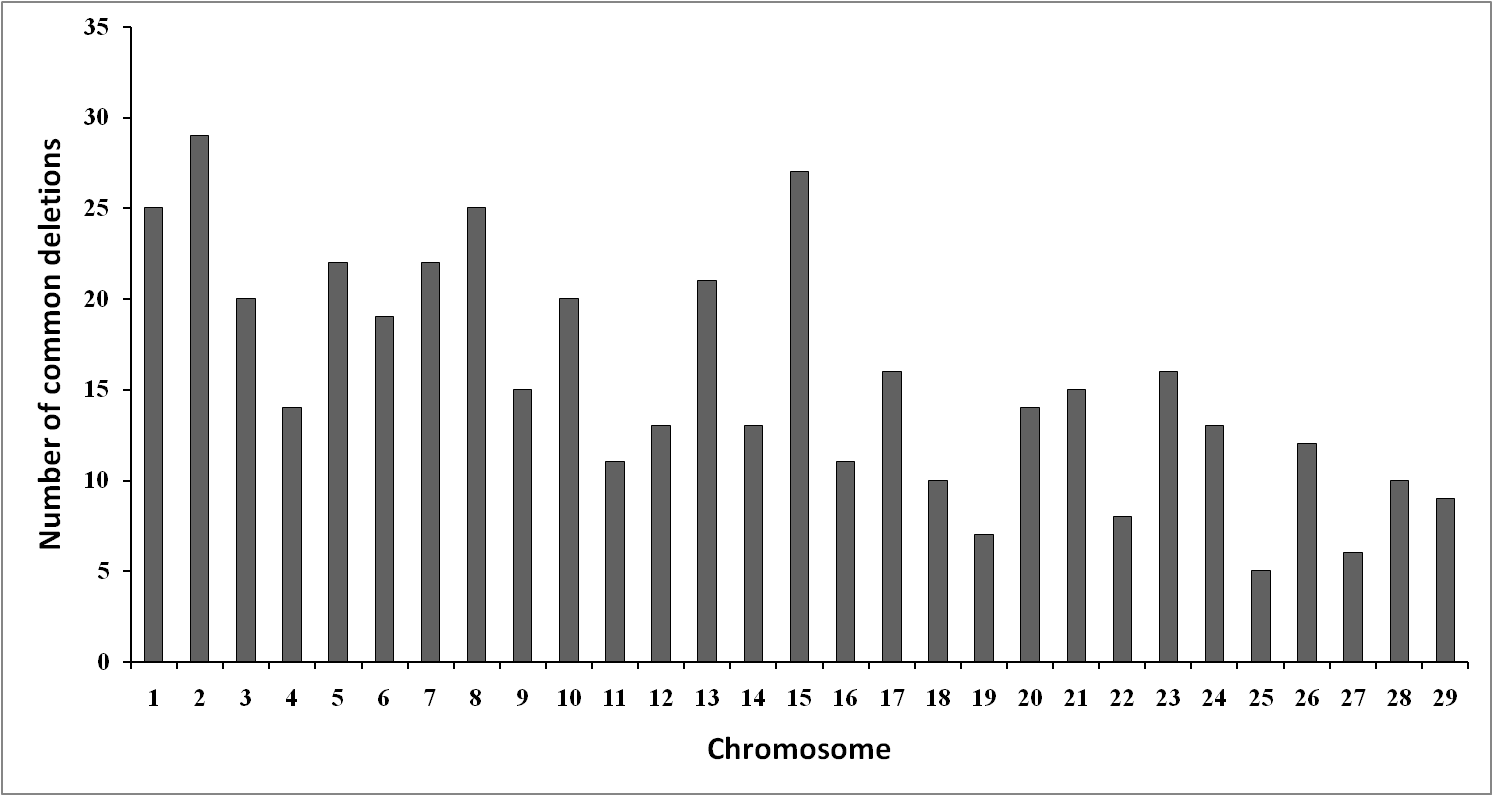


Supplementary Figure 4

Supplementary Figure 5
